# Pediatric kidney transplant recipients with and without underlying structural kidney disease have a comparable risk of hospitalization associated with urinary tract infections

**DOI:** 10.3389/fped.2022.953139

**Published:** 2022-09-02

**Authors:** Elizabeth Spiwak, Corina Nailescu, Andrew Schwaderer

**Affiliations:** ^1^Division of Pediatric Nephrology, Peyton Manning Children’s Hospital, Indianapolis, IN, United States; ^2^Division of Pediatric Nephrology, Department of Pediatrics, Indiana University, Indianapolis, IN, United States

**Keywords:** pyelonephritis, immunosuppression, congenital anomalies of the kidney and urinary tract (CAKUT), allograft, *E. coli*

## Abstract

**Introduction:**

Urinary tract infections (UTIs) are a common and potentially serious kidney transplant complication. Pediatric kidney transplants are potentially at increased risk for UTIs when structural kidney disease is the underlying end-stage kidney disease (ESKD) etiology. The objective of this manuscript is to determine if children with structural kidney disorders are more prone to UTIs post kidney transplant.

**Materials and methods:**

Hospitalizations for pediatric kidney transplant recipients were retrospectively reviewed over a 4-year period for UTIs in the diagnostic codes. The patient’s age, sex, graft age, underlying diagnosis for cause of ESKD, symptoms at presentation, urinalysis results, and urine culture results were recorded. UTI rates, febrile UTI rates, and UTI rates in the 1st year post-transplant were compared between children with ESKD due to structural vs. non-structural kidney disease.

**Results:**

Overall, 62 of 145 pediatric patients with kidney transplants accounted for 182 hospitalizations for kidney transplant complications over the 4-year study period. UTIs were components of 34% of the hospitalizations. Overall, UTI rates, febrile UTI rates, and UTI rates for the 1st year post kidney transplant were comparable for children with vs. without structural ESKD etiologies.

**Conclusion:**

Urinary tract infections are frequent components of hospitalizations for pediatric kidney transplant recipients. Children with and without structural kidney disease as an ESKD etiology have similar UTI rates indicating that UTI susceptibility is primarily due to the transplant process and/or medication regimens. UTIs represent a potentially modifiable risk factor for pediatric kidney transplant complications.

## Introduction

According to the United Network for Organ Sharing (UNOS), almost 650 pediatric patients (less than 18 years of age) received a kidney transplant in 2020 (1). While kidney transplants can improve quality of life, medication burden, and length of life, patients with kidney transplants are at increased for a range of infections. Urinary tract infections (UTIs) are well-established complications of kidney transplantation, particularly when they ascend into the graft resulting in transplant pyelonephritis (2). UTIs are the most common infection experienced by kidney allograft recipients, occurring at a rate of 36–48% during the course of the allograft (3–5). In other patient populations at risk for UTIs including children with vesicoureteral reflux and post-menopausal women, strategies have been defined to limit UTIs in “at risk” patients (6). Despite UTIs being a well-known complication of kidney transplants, “at risk” subpopulations of pediatric patients with kidney transplants to target for interventions have not been defined. Therefore, the scope of UTIs in children with kidney transplants warrants investigation as a potentially preventable complication.

Congenital anomalies of the kidney and urinary tract (CAKUT), by way of obstruction and vesicoureteral reflux (VUR), among others, are a known risk factor for the development of UTIs in the general population (7). Risk factors for UTI in CAKUT patients are likely due to urine stasis from suboptimal drainage and/or retrograde flow of urine from the bladder to the native kidneys and/or transplant graft resulting in vesicoureteral reflux (8).

The objective of this study was to review the hospitalizations of pediatric patients with kidney transplants and determine if UTI-related admissions were concentrated in children with end-stage kidney disease (ESKD) from CAKUT.

## Materials and methods

This study was a retrospective chart review and was approved by the Indiana University Institutional Review Board, protocol # 2003884720. Charts were obtained from pediatric kidney transplant recipients who were transplanted between 01/01/2000 and 12/31/2019 at the Riley Hospital for Children/Indiana University Department of Pediatrics. This list was cross-referenced with one created from all patients admitted to the hospital between 01/01/2016 and 12/31/2019 with any of the following ICD 10 codes: Z94.0 (kidney transplant status), T86.10 (an unspecified complication of kidney transplant), T86.13 (kidney transplant infection), T86.19 (other complication of kidney transplant), N10 (acute pyelonephritis), N39 (urinary tract infection, site not specified), and N17 (acute kidney failure). Patients who had less than 1-year of follow-up or who had multiple organs transplanted were excluded. Each hospital admission was reviewed, and a spreadsheet was created with the patient’s age, sex, graft age, underlying diagnosis for cause of ESKD, symptoms at presentation, urinalysis results, urine culture results, and baseline serum creatinine as an outpatient prior to admission, admission serum creatinine, and admission serum bicarbonate. A UTI diagnosis was defined by the patient being diagnosed and treated for a UTI during the hospitalization and having bacteria with a concentration of at least 10^3^ CFU/ml isolated on urine culture. We defined pyuria as having at least 1 + leukocyte esterase (LE) and/or 6 WBCs on urinalysis. Allograft rejection was assigned to patients who were diagnosed with cellular and/or antibody-mediated rejection on kidney biopsy.

### Immunosuppression and infection prophylaxis

Immunosuppression consisted of antithymocyte globulin (rabbit) thymoglobulin and methylprednisolone start was started perioperatively followed by four daily post-operative doses. Mycophenolate mofetil 430–600 mg/m^2^ PO twice daily was started on postoperative day 0 and tacrolimus 0.15 mg/kg/dose q12h was generally started on postoperative day 1. Initial goal tacrolimus levels were 7–10 ng/ml. In select cases, sirolimus and/or corticosteroids were used in addition or instead of tacrolimus or mycophenolate mofetil. Pneumocystis pneumonia prophylaxis consisted of sulfamethoxazole/trimethoprim given at 3–5 mg/kg day for 6 months (maximum 80 mg trimethoprim started on post-op day 4. In the case of sulfa-drug allergy pentamidine was used and 9 mg/kg (maximum 300 mg) inhalation monthly for age 5 years or above and 4 mg/kg/dose iv monthly for patients below the age of 5 years. Methylprednisolone 4 mg/kg IV X 1 (maximum 250–500 mg). There was no standard UTI monitoring outside of urinalysis at clinic visits and as-needed urinalysis and urine cultures for UTI symptoms.

### Data analysis

Data analysis was performed with GraphPad Prism (GraphPad Software, San Diego, CA, United States). Data were tested for normality using the Shapiro-Wilk test. Two groups were compared with the *t*-test (2 tailed) or Mann-Whitney for parametric and non-parametric data respectively. More than two groups were compared with a one-way ANOVA or Kruskal-Wallis for parametric and non-parametric data respectively. Percentages were compared with the chi-square test, or if the expected frequency was <5, the Fisher exact test was used instead.

## Results

### Included patients and demographics

A total of 68 of 145 (47%) children with kidney transplants were hospitalized post-transplant for complications. Exclusion criteria were present in six patients; five were followed for less than 1 year and one had a concurrent heart transplant *en bloc* with kidneys. The remaining 62 patients accounted for 182 individual hospital visits. The demographics of the included patients subdivided between those with CAKUT vs. non-CAKUT underlying ESKD etiologies are outlined in Table 1. The CAKUT group had a higher percentage of men, was younger, and was more likely to be undergoing a chronic bladder catheterization program than the non-CAKUT group. Nephrectomies of the native kidneys were uncommon only occurring in three patients.

**TABLE 1:** Patient demographics and background.

	CAKUT	Non-CAKUT	*P*-value
Patients	38	24	
Male:female (% male)	33:5 (87%)	11:13 (46%)	0.001
Age at transplant	8.13 ± 4.80 (86%)	11.42 ± 4.37 (46%)	0.013
Underlying diagnosis	Obstructive nephropathy (ON): 18^a^ Refluxing-non-ON: 5^b^ Dysplasia: 15^c^	Acquired kidney injury: 5^d^ Glomerular disease: 10^e^ Genetic PCKD, ciliopathy or tubulopathy: 9^f^	
Hospitalizations	124 (3.3 per patient)	58 (2.41 per patient)	
Chronic bladder catheterization	14^g^	0	<0.001
Nephrectomies of native kidneys	2	1	>0.999
Time period followed	5.34 ± 3.27	6.08 ± 4.09	0.4428

^a^Posterior urethral valves (12), prune belly syndrome (2), meatal stenosis (1), neurogenic bladder (1), other obstructive uropathy (2).

^b^Reflux nephropathy (1) solitary dysplastic kidney with vesicoureteral reflux (VUR [1]) and with bilateral dysplastic kidneys with reflux (3).

^c^Bilateral cystic dysplasia (4), bilateral renal dysplasia (9), solitary dysplastic kidney (2).

^d^Cortical necrosis (3) and bilateral Wilm’s tumor post resection (2).

^e^Alport’s syndrome (2), focal segmental glomerulosclerosis (3), membranoproliferative glomerulonephritis (1), lupus nephritis (1), IgA nephropathy (1), vasculitis (1), congenital nephrotic syndrome (1).

^f^Nephronopthisis (5), autosomal dominant polycystic kidney disease (PCKD) (1), autosomal recessive PCKD (2), cystinosis (1).

^g^All patients requiring bladder catheterization were in the obstructive nephropathy (ON) subgroup.

### Hospitalized urinary tract infections rates and characteristics between congenital anomalies of the kidney and urinary tract and non-congenital anomalies of the kidney and urinary tract patients

Overall, 22/62 (35%) of hospitalized pediatric patients with kidney transplants had a documented UTI with a positive urine culture. UTI rates and characteristics for the CAKUT vs. non-CAKUT groups are presented in Table 2. UTIs accounted for 55/128 (30%) of hospitalizations and occurred at increased frequencies than hospitalizations for kidney allograft rejection. The percentages of patients with UTIs, UTIs occurring in the first post-transplant year, hospitalizations for UTIs, hospitalizations for kidney allograft rejection, or admissions with non-UTI bacterial infections were not significantly different between the CAKUT and non-CAKUT groups. Of the UTI hospitalizations, rates of febrile UTIs, urosepsis and *Escherichia coli* isolation on urine culture were not different between groups. A subgroup analysis of the CAKUT group (Table 3) demonstrated that there were no significant differences in UTI rates or hospitalizations with UTIs between CAKUT patients with obstructive nephropathy, vesicoureteral reflux or non-refluxing, non-obstructive nephropathy. Among the obstructive nephropathy subgroup, there were no differences in UTI rates between patients that were on a chronic bladder catheterization vs. those who were not. A subgroup analysis of non-CAKUT patients (Table 4) demonstrated no significant differences in UTI rates or hospitalizations with glomerulopathies/vasculitis, genetic polycystic kidney disease (PCKD)/ciliopathies/tubulopathies or those with acquired kidney injury.

**TABLE 2:** Congenital anomalies of the kidney and urinary tract (CAKUT) vs. non-CAKUT, urinary tract infection (UTI) rates and characteristics.

	CAKUT	Non-CAKUT	*P*-value
Patient with UTIs	14/38 (37%)	8/24 (33%)	0.790
UTIs in 1st year	4/38 (11%)	2/37 (8%)	>0.999
UTIs per year	0.19 ± 0.51	0.33 ± 0.77	>0.994
Hospitalizations with UTIs	36/124 (27%)	19/58 (33%)	0.6081
Hospitalizations with graft rejection	23/124 (19%)	7/58 (12%)	0.391
Hospitalizations with non-UTI bacterial infections	2/124^a^ (2%)	4/58^b^ (7%)	0.083
Time period from transplant to UTI hospitalizations	4.20 ± 3.47	5.63 ± 5.04	0.222
UTI admissions with febrile UTIs	18/36 (50%)	9/19 (47%)	>0.999
UTI admissions with urosepsis	2/36 (6%)	2/19 (11%)	0.602
UTI admissions with *E. coli* isolated on urine culture	13/36 (36%)	9/19 (47%)	0.401

^a^Pneumonia (1), osteomyelitis (1).

^b^Abdominal abscess (1), pneumonia (1), shigella diarrhea (1), cellulitis (1).

**TABLE 3:** Congenital anomalies of the kidney and urinary tract (CAKUT) group sub-analysis: urinary tract infection (UTI) rates and characteristics.

	Obstructive nephropathy (*n* = 20)	Non-obstructive, non-refluxing dysplasia (*n* = 13)	Primary vesicoureteral reflux (5)	*P*-value
Age at transplant (years)	8.64 ± 4.89	7.48 ± 4.70	7.80 ± 4.87	0.794
Male:female	20:0	10:3	3:2	0.039^a^
Follow-up time (years)	4.66 ± 2.58	5.41 ± 3.33	7.87 ± 4.86	0.140
Patients with UTIs	8/20^b^	4/13	1/5	0.667
UTIs per year	0.15 ± 0.24	0.29 ± 0.75	0.12 ± 0.27	0.814
Hospitalizations with UTIs	18/69 (26%)	16/44 (36%)	2/11 (18%)	0.356
% febrile UTIs	10/18 (55%)	8/16 (50%)	1/2 (50%)	0.948
UTIs with *E. coli*	8/18 (44%)	4/16 (25%)	1/2(50%)	0.270

^a^Males are a significantly higher proportion in the obstructive nephropathy group compared to the primary VUR or non-obstructive/non-refluxing dysplasia groups.

^b^5/13 (38%) ON patients undergoing a chronic catheterization program had a UTI vs. 3/7 (42.3%) of ON not on a catheterization program, *p* = 0.999.

**TABLE 4:** Non-congenital anomalies of the kidney and urinary tract (CAKUT) group sub-analysis: urinary tract infection (UTI) rates and characteristics.

	Glomerulopathy/vasculitis (*n* = 10)	Genetic PCKD, ciliopathy or tubulopathy (*n* = 9)	Acquired kidney injury (*n* = 5)	*P*-value
Age at transplant (years)	14.42 ± 1.86	10.4 ± 3.21	6.28 ± 4.85	0.008^a^
Male:female (% male)	3:7 (30%)	6:3 (66%)	3:2 (60%)	0.247
Follow-up time (years)	5.32 ± 1.85	6.34 ± 5.42	7.82 ± 4.90	0.457
Patients with UTIs	5/10 (50%)	3/9	0/5	0.153
UTIs per year	0.46 ± 0.72	0.38 ± 1.00	0.00 ± 0.00	0.155
Hospitalizations with UTIs	12/30 (40%)	7/19 (37%)	0/9 (0%)	0.072
% febrile UTIs	5/12 (42%)	5/7 (71%)	N/A	0.350
UTIs with *E. coli*	5/12 (42%)	3/7 (43%)	N/A	>0.999

^a^Acquired kidney injury has significantly lower age than glomerulopathy/vasculitis with an adjusted *p*-value of 0.014.

### Hospitalized urinary tract infections rates and characteristics between men and women patients

Patient characteristics and UTI rates were compared between men and women patients (Table 5). There were no significant differences in the percentage of men vs. women patients who had a UTI. Women patients did have a higher rate of hospitalizations for UTIs than men patients.

**TABLE 5:** Male vs. female: urinary tract infection (UTI) rates and characteristics.

	Male (*n* = 44)	Female (*n* = 18)	*P*-value
Age at transplant (years)	8.92 ± 4.99	10.32 ± 4.45	0.304
Follow-up time (years)	5.58 ± 3.30	7.06 ± 4.06	0.052
Patients with UTIs	14/44 (31.2%)	8/18 (44%)	0.399
UTIs per year	0.23 ± 0.64	0.30 ± 0.58	0.247
Hospitalizations with UTIs	35/135 (26%)	20/47 (45%)	0.042
% febrile UTIs	19/35(54%)	9/20 (45%)	0.582
UTIs with *E. coli*	7/19 (37%)	11/20 (55%)	0.346

### Urinary tract infection vs. non-urinary tract infection hospitalizations

Admission characteristics and laboratory findings were compared between UTI and non-UTI hospitalizations (Table 6). There was a trend toward a higher admission serum creatinine compared to pre-admission baseline and length of hospitalization in the UTI admissions vs. the non-UTI admissions, which just missed the significance. The patients with UTIs were more likely to have pyuria, nitrites in the urine, and a lower admission serum bicarbonate.

**TABLE 6:** Comparison of urinary tract infection (UTI) admissions vs. non-UTI admissions.

	UTI admission (*n* = 55)	Non-UTI admission (127)	*P*-value
Length of admission (days)	5.38 ± 4.77	4.44 ± 3.99	0.059
Time from transplant to admission (years)	4.69 ± 4.09	3.74 ± 3.00	0.2732
Fever as presenting symptom	28/55 (51%)	33/127 (26%)	0.002^a^
Pyuria^b^	52/55 (95%)	29/127 (72%)	<0.001^c^
≥1 + nitrites on UA	13/55 (24%)	0/127 (0%)	<0.001^d^
Δ serum creatinine (baseline to peak hospitalization [mg/dl])	+0.62 ± 0.95	+0.50 ± 1.01^e^	0.079
Admission serum bicarbonate (mEq/l)	18.7 ± 3.8	20.5 ± 3.3	0.013

^a^The odds ratio for a UTI when fever is the presenting symptom was 3.0 (95% CI of 1.3–3.2) sensitivity 51% (95% CI of 38–63%), specificity 74% (95% CI 34–58%).

^b^≥1 + leukocyte esterase and/or ≥ 5 WBCs on UA.

^c^The odds ratio for a UTI when pyuria was present was 58.6 (95% CI of 17.6–184.9), sensitivity of 95% (95% CI of 85–99%), and specificity of 74% (95% CI 69–84%).

^d^The odds ratio had a 95% CI of 9.5 to infinity, sensitivity of 23% (95% CI of 14–36%) and specificity of 100% (95% CI 97–100%).

^e^A serum creatinine was not checked in 1 non-UTI admission.

### Urine culture isolates

Urine culture isolates are presented in Table 7. *Escherichia coli* (*E. coli*) was the most common isolate followed by *Klebsiella*, *Enterobacter*, and *Enterococcus* species. Four patients had multiple isolates on culture. One non-CAKUT patient had a single bacteria species on an initial culture, but a follow-up culture grew two isolates, which included the species of the initial isolate but at a much lower concentration. A second non-CAKUT patient grew both *E. coli* and *Klebsiella* pneumonia on urine culture, but was treated as a UTI because they presented with a fever and dysuria, had >100 WBCs, and positive nitrites on urinalysis. A third non-CAKUT patient grew both *Klebsiella oxytoca* and *S. saprophyticus* and was treated as a UTI because they presented with a fever, vomiting, and 50–100 WBCs on urinalysis. A CAKUT patient with non-refluxing, non-obstructing dysplasia grew *Globicatella* species and *Providencia rettgeri* but was diagnosed and treated as a UTI because they presented with graft pain and 50–100 WBCs on urinalysis. Two patients had culture results with concentrations < 50^4^ CFU/ml, the aforementioned repeat isolate with a lower concentration and the CAKUT patient that had both *Globicatella* species and *P. rettgeri* isolated on urine culture.

**TABLE 7:** Urine culture isolates.

Isolate	Total (% of positive cultures, *n* = 55)
*Escherichia coli*	23 (42%)
*Klebsiella* species	12 (22%) [pneumonia (7), oxytoca (3), unspecified (2)]
*Enterobacter* species	5 (9%) [cloacae (4), aerogenes (1)]
*Enterococcus* species	4 (7%) [faecium (2), unspecified (2)]
*Streptococcus* species	3 (5%) [viradians (1), agalectaie (1), unspecified (1)]
*Staphylococcus* species	3 (5%) [aureus (2), saprophyticus (1)]
*Pseudomonas aeruginosa*	3 (5%)
*Providencia rettgeri*	2 (4%)
*Acinobacter baumannii*	2 (2%)
*Globicatella* species	1 (2%)
*Raoultella ornithinolytica*	1 (2%)
*Citrobacter freundi*	1 (2%)
*Achromobacter xylosoxidans*	1 (2%)

## Discussion

Despite the known risk of UTI with CAKUT, this study reveals the risk of UTI post-transplantation is similar among all types of underlying end-stage kidney disease etiologies (9). Thus, the process of transplantation and/or resultant immunosuppression appears to be an independent risk factor for UTIs.

The most common organism resulting in UTI in the pediatric kidney transplant recipients analyzed was *E. coli*. However, *E. coli* only accounted for 42% of isolates in this case series, which is lower than the ∼80–90% *E. coli* rate for children with UTIs overall (10). Thus, non-*E. coli* UTIs appeared to occur with increased frequency in the pediatric transplant population. The organism profile in our patients is consistent with previous studies where the predominant species noted for both adult and pediatric patients with kidney transplants diagnosed with early UTIs were *Klebsiella pneumoniae* and *E. coli;* the variety of uropathogens in patients with kidney transplants has been proposed to relate to immunosuppressive medications, bacterial colonization, antibiotic prophylaxis, and bladder malformations (11).

In febrile children aged 2–24 months at least 50^4^ colony-forming units per milliliter (CFUs/mL) is considered the threshold for UTI diagnosis, however, there is some discussion about lowering this threshold to 10^4^ CFU/ml (12). In patients with a kidney transplant, 10^3^–10^5^ CFU/ml have been used as thresholds for UTIs (13, 14). After an extensive search of the literature, we could not find that any of these thresholds have been validated to apply to the kidney transplant populations. Patients with kidney transplants may be more likely to grow atypical organisms that might not grow as well under standard urine infections. To this end, recent UTI guidelines for solid organ transplants published by Goldman and Julian in recommend that >10^3^ CFU/ml and > 10 WBC/mm be used as the threshold for cystitis and >10^4^CFU/ml and >10 WBC/mm be used to diagnose pyelonephritis (15). Future prospective studies using expanded urine culture conditions and 16S sequencing should be considered with the objective of identifying bacteria identification patterns that represent dysbiosis. We did not track the culture collection method, but a catheter or midstream clean catch sample using the proper technique is important to limit contaminated cultures. We did include some patients with multiple organisms on cultures that had clinical presentations and courses consistent with UTIs. Multiple organisms raise the possibility of contamination, but like the urine isolate concentration threshold, how to address multiple isolates remains to be defined in the kidney transplant population (16). A UTI is considered complicated if the infection is present in a patient with structural or functional abnormalities (16). In our study, we found that the rate of UTI incidence was the same regardless of whether the patient had an underlying structural etiology. This indicates that the transplant process and/or medication environment itself presents the primary risk factor for infection in this population rather than the underlying end-stage kidney disease etiology. We do note that the non-CAKUT group had a higher percentage of women patients which might have skewed these results because, outside of the initial year of life, women are more susceptible to UTIs.

Potential risk factors for UTIs in all patients with kidney transplants include immunosuppression or anatomical risk from the transplant procedures. Immunosuppression for pediatric kidney transplantation at Indiana University generally involves induction with anti-thymocyte globulin followed by chronic immunosuppression with mycophenolate mofetil and the calcineurin inhibitor tacrolimus for the duration of the time period studied in this report. Corticosteroids and rapamycin are used in some patients as well. Mycophenolate mofetil has been associated with UTIs when used in other populations such as adults with retroperitoneal fibrosis (17). In mice, tacrolimus has been shown to increase UTI susceptibility (18). Whether alternative immunosuppression such as corticosteroids and/or rapamycin or dose adjustments in mycophenolate mofetil or tacrolimus would lessen UTI risk in pediatric kidney transplant warrants investigation in future studies. Prior studies have found the presence of VUR in the native kidneys prior to transplantation to increase the risk of UTI in adult and pediatric renal transplant recipients (19, 20). However in most cases of non-structural renal disease, a pretransplant VCUG would not be performed. Allograft VUR may be associated with febrile UTIs in some patients, but its disease relevance in pediatric kidney transplants remains to be defined (21). Would correcting the reflux surgically prior to transplantation provide protection for that post-transplantation risk? Basiri et al. (22) found that those who had surgical correction of VUR continued to have a higher risk for UTI post-transplant than those recipients who had no history of VUR.

Other studies have evaluated the underlying kidney disease relative to UTI risk following pediatric kidney transplantation. Fallahzadeh et al. (23) evaluated the UTIs in the 1st month after a kidney transplant. UTIs occurred in 24/148 (17%) patients and half of the patients with UTIs experienced ≥ 2, but significant differences were not present based on primary kidney disease. Monteverde et al. (24) evaluated 923 patients over two eras (1988–2000 and 2001–2009). Twenty-nine percent of patients with CAKUT vs. 17% of patients without CAKUT experienced a post kidney transplant UTI. Our results are more consistent with the results of Fallahzadeh et al. (23). While our sample size was smaller than Monteverde et al. (24), we also controlled for the number of UTIs per year and performed a sub-analysis to highlight febrile UTI rates. Febrile UTIs are more of a concern because they raise the potential of the UTI progressing from the lower urinary tract into the allograft, native kidneys, or even the bloodstream (25). Although it should be noted that a fever may be muted in immunosuppressed patients (26).

Strategies have been developed to limit UTI risk in other patient populations such as antibiotic prophylaxis for children with vesicoureteral reflux (6). The pediatric patients with kidney transplants at our center are treated with sulfamethoxazole and trimethoprim for 6 months for pneumocystis pneumonia prophylaxis. We do not routinely use UTI prophylaxis. Some of the patients undergoing chronic bladder catheterization programs do undergo bladder irrigations with antibiotics. Of note, patients with obstructive nephropathy did not experience higher rates of UTI compared to patients without obstructive uropathy. Testing the safety and efficacy of antibiotic prophylaxis in pediatric patients with kidney transplants represents a future research direction. In the “Urinary tract infections in solid organ transplant recipients: Guidelines from the American Society of Transplantation Infectious Diseases Community of Practice” strategies to limit UTIs and/or UTI recurrence include reminding patients of basic infection prevention measures (e.g., hydration, frequent/timed voiding, toilet hygiene, voiding after sexual intercourse, and avoidance of sequential anal and vaginal intercourse), topical vaginal estrogen for post-menopausal women, identifying and addressing any structural UTI risk factors, antimicrobial prophylaxis, removal of ureteral stents if present and vigilance for donor-derived infections) (15).

This study does have multiple limitations. The men:women ratio was not the same so sex differences between the CAKUT and non-CAKUT groups may have influenced the results. Another limitation is that we grouped prune belly syndrome, renal dysplasia, and VUR, among others together as “structural abnormalities” and did not analyze VUR into the allograft as a UTI risk post-transplantation. Also, we included patients that the treating physicians diagnosed with UTIs which in some cases had cultures results that were positive for multiple organisms. In a non-transplant child, this culture result might not be considered consistent with a UTI; however, in transplanted patients, the criteria for positive urine cultures are less well-defined. No patients had a DMSA scan, the gold standard imaging to identify pyelonephritis, probably because this study is often not practical to obtain in the acute setting (27). For future prospective studies contrast-enhanced ultrasound might be a helpful modality to confirm the diagnosis of acute pyelonephritis (28). Another limitation is the population size. Since this was a one-center chart review of hospitalized patients, and only over a 5-year time period, the population studied only consisted of 62 patients. Some UTI risk factors such as sexual activity or constipation were not included in this analysis due to the retrospective study design. Additionally, animal model studies might be needed to lend insight into the mechanisms for UTI susceptibility including immunosuppression, allograft VUR, and VUR into native kidneys.

The technique of implanting the graft ureter into the recipient’s bladder (that can be either an antireflux technique, which carries the risk of strictures, or a non-antireflux technique, which will be associated with vesicoureteral reflux) the use of stents to prevent leaks and complications, as well as the use of urinary catheters in the post-transplant period can all contribute to the risk of UTI in a patient with the transplant (29).

In conclusion, we demonstrate that post-transplant UTIs occur in approximately a third of pediatric kidney transplants who are hospitalized. Overall, UTI and febrile UTI rates were not statistically different between children with underlying ESKD from structural vs. non-structural kidney disease. Also, non-*E. coli* UTIs appear to be more frequent in post-transplant UTIs. UTI prevention in patients with pediatric kidney transplants represents a potential clinical target for improved outcomes in children who receive kidney transplants.

## Data availability statement

The raw data supporting the conclusions of this article will be made available by the authors, without undue reservation.

## Ethics statement

This study was a retrospective chart review and was approved by the Indiana University Institutional Review Board, protocol # 2003884720. Written informed consent from the participants’ legal guardian/next of kin was not required to participate in this study in accordance with the national legislation and the institutional requirements.

## Author contributions

AS and ES designed the study. ES performed data analysis and wrote the first draft. CN did data analysis and performed a critical review of the manuscript. AS supervised the study. All authors contributed to the article and approved the submitted version.
